# Utilization of post-exposure prophylaxis potentially contributed to the changes of risk behaviors among men who have sex with men in China

**DOI:** 10.3389/fpubh.2024.1364913

**Published:** 2024-04-08

**Authors:** Rong Su, Yi Liu, Peilong Li, Lin Ge, Meizhen Liao, Yong Fu, Xin Song, Houlin Tang, Dongmin Li

**Affiliations:** ^1^National Center for AIDS/STD Control and Prevention, Chinese Center for Disease Control and Prevention, Beijing, China; ^2^Shandong Provincial Center for Disease Control and Prevention, Jinan, China; ^3^Qingdao Municipal Center for Disease Control and Prevention, Qingdao, China

**Keywords:** men who have sex with men, HIV, post-exposure prophylaxis, utilization, risk behaviors

## Abstract

**Background:**

The HIV infection status among men who have sex with men (MSM) in China is a cause for concern. Post-exposure prophylaxis (PEP) serves as a highly effective biomedical preventive measure against HIV infection. Substantial evidence has established an association between PEP utilization and risk behaviors among MSM, but whether the utilization of PEP has an impact on risk behaviors remains unknown. This study sought to elucidate the impact of PEP usage on risk behaviors among MSM and provide recommendations for developing targeted HIV prevention programs.

**Methods:**

A cohort study was conducted in Qingdao, China, from April 2021 to January 2022. Participants were enlisted by volunteers from community-based organizations through a snowball sampling method. Face-to-face interviews were conducted to collect sociodemographic and behavioral information of participants. The study encompassed a retrospective investigation, baseline survey, and follow-up survey, representing periods before, during, and after PEP usage, respectively. Generalized estimating equations, fitting a Poisson regression model, were applied to scrutinize changes in risk behaviors of MSM during and after PEP usage, in comparison to before PEP usage.

**Results:**

A total of 341 MSM were recruited in the cohort study, with 179 individuals completing the follow-up survey. In comparison to before PEP usage, there was a significant increase in the proportion of Rush Popper usage (17.6% vs. 23.8% vs. 29.6%) and commercial sexual partners (10.9% vs. 17.6% vs. 21.8%) among MSM during and after PEP usage. Before PEP usage, 88.7% of MSM reported having ≥3 temporary sexual partners in the last 6 months. This proportion exhibited no significant change during PEP usage (91.8%), but it significantly increased to 97.8% after PEP usage (P < 0.05). Notably, there was a significant decrease in group sex during and after PEP usage compared to before PEP usage (30.8% vs. 21.4% vs. 21.2%).

**Conclusion:**

The utilization of PEP may impact risk behaviors among MSM, potentially leading to increased Rush Popper usage, temporary sexual partners, and commercial sexual partners after PEP usage, accompanied by a decrease in group sex. Further research is imperative to elucidate the impact of PEP utilization on MSM and develop targeted HIV prevention programs.

## Introduction

Men who have sex with men (MSM) have always constituted a key population for HIV infection due to risk behaviors, such as recreational drug use, participation in group sex, and engaging in unprotected anal sex (1). Globally, the median HIV prevalence among MSM was 7.5%, significantly surpassing that of general adult population aged 15–49 years, which was 0.7% (2). The HIV prevalence in China remains relatively low, with the majority of newly reported HIV infections in recent years attributed to sexual behavior. Notably, the proportion of HIV transmission through homosexual sex has seen a substantial increase, escalating from 2.5% in 2006 to 25.6% in 2022 (3). Among MSM in China, the HIV prevalence has reached 7.0%, with certain regions exceeding 20.0%, indicating a concerning HIV epidemic within this population (4).

Initially, the HIV prevention and control measures for MSM in China primarily focused on health education, promoting condom use, and encouraging regular HIV testing. These measures have achieved notable success, contributing to significant improvements in HIV knowledge and testing rates among MSM. However, challenges such as condom-related stigma (5), sexual sensation (6), and risk perception (7) have resulted in only about half of MSM in China consistently using condoms during sexual activities, implying a persistent high risk of HIV infection in this population. Therefore, in order to achieve the goal of ending the AIDS epidemic by 2030, the promotion of biomedical preventive measures is indispensable.

Post-exposure prophylaxis (PEP) is a biomedical preventive measure used to block HIV infection following exposure, with its efficacy substantiated in numerous studies (8, 9). Initially employed for occupational post-exposure prevention (10), PEP expanded to non-occupational settings with the spread of HIV (11). Currently, multiple guidelines on PEP recommend individuals at risk of HIV infection to initiate PEP for prevention within 72 hours of HIV exposure (12–14). The global promotion and application of PEP have spurred its demand among MSM in China. In 2016, Chinese Center for Disease Control and Prevention (CDC) designated PEP as a crucial measure for HIV prevention among MSM (15). MSM can access PEP through diverse channels, including hospitals, pharmacies, and Blued app (a MSM social media).

Substantial evidence has established an association between PEP utilization and risk behaviors among MSM (16–18). The engagement in risk behaviors prompts MSM to use PEP, yet whether the utilization of PEP has an impact on risk behaviors of MSM remains unknown. A cohort study was conducted among MSM using PEP in Qingdao from 2021 to 2022, with the objective of comprehending the impact of PEP usage on the risk behaviors of MSM. The findings aim to provide recommendations for the development of targeted HIV prevention measures.

## Materials and methods

### Study design and participants

This cohort study conducted in Qingdao comprises three components: a baseline survey, a retrospective investigation, and a follow-up survey. MSM aged 18 years and above, residing in Qingdao, who were HIV-negative, and utilized PEP in the last 6 months were recruited through a snowball sampling method. Between April and August 2021, volunteers from local community-based organizations (CBOs) recruited eligible MSM to conduct the baseline and retrospective survey. During this period, participants were asked to recommend individuals around them to participate. Recruitment took place in diverse venues such as bars, bathhouses, and parks, etc. The follow-up survey was conducted 6 months later. During this period, CBOs stayed in touch with study participants via WeChat, encouraging their engagement in the follow-up.

To improve the cooperation and data quality, this study employed volunteers from local CBOs as investigators. These volunteers maintained close connections with the local MSM community, resulting in increased cooperation during the investigation. Comprehensive training sessions were uniformly provided to all investigators, ensuring the acquisition of specific investigation skills. On-site surveys were conducted in separate rooms to safeguard the privacy of participants.

### Measures

All data were collected through face-to-face interviews. The following information was collected: sociodemographic characteristics (age, marital status, registered residence, education, occupation, income, and sexual orientation), Rush Popper usage in the last 6 months (how to use, where to use, how often to use), sexual behaviors in the last 6 months (temporary sexual partners, commercial sexual partners, unprotected anal sex, group sex, etc.), HIV infection status, PEP usage.

All participants had utilized PEP in the last 6 months during the baseline survey, categorizing this phase as during PEP usage. Subsequently, the retrospective and follow-up surveys were designated as before and after PEP usage, respectively.

### Statistical analysis

Two researchers independently entered and verified the raw data using EpiData3.1 software and created a database. Statistical analyses were performed using R4.2.3 software. Descriptive analyses were conducted to describe the sociodemographic characteristics of study participants. The chi-square test was employed to compare sociodemographic differences between followers and those lost to follow-up. Outcome variables in this study encompassed various risk behaviors potentially facilitating HIV transmission, such as Rush Popper usage, temporary sexual partners, commercial sexual partners, group sex, and so on. Generalized Estimating Equations fitting a Poisson regression model, were used to analyze changes in outcomes during and after PEP usage compared to before PEP usage. Comparing during PEP with before PEP sought to discern the possible short-term impact of PEP utilization on MSM. Comparing after PEP with before PEP aimed to understand the stable impact of PEP utilization on risk behaviors among MSM. Variables with statistical significance were further stratified by sociodemographic characteristics. All statistical tests were two-sided and were statistically significant at *P* < 0.05.

## Results

### Study population

In the cohort study, a total of 341 MSM were recruited, with 179 of them completing the follow-up survey. Both the follow-up and loss of follow-up groups predominantly consisted of individuals aged 19–29 years, unmarried, registered in Qingdao, holding a college degree or higher, employed full-time, earning an average monthly income of ≥5,000 RMB, and identifying with a homosexual orientation. Notably, statistical differences were observed in age and average monthly income between the two groups, while the other characteristics showed no statistically significant differences (Table 1).

**Table 1:** Comparison of sociodemographic characteristics between follow-up and loss to follow-up groups.

**Characteristics**	**Follow-up**	**Loss to follow-up**	**X^2^**	***P***
**Age group**			7.686	0.021
19–29	81 (45.3%)	95 (58.6%)		
30–39	76 (42.5%)	46 (28.4%)		
≥40	22 (12.3%)	21 (13.0%)		
**Marital status**			0.022	0.881
Unmarried	136 (76.0%)	121 (74.7%)		
Married/divorced/widowed	43 (24.0%)	41 (25.3%)		
**Registered residence**			0.010	0.919
Qingdao	118 (65.9%)	105 (64.8%)		
Others	61 (34.1%)	57 (35.2%)		
**Education**			2.354	0.125
High school or lower	35 (19.6%)	44 (27.2%)		
College or higher	144 (80.4%)	118 (72.8%)		
**Whether having a full-time job**			< 0.001	1.000
Yes	153 (85.5%)	138 (85.2%)		
No	26 (14.5%)	24 (14.8%)		
**Average monthly income**			24.630	< 0.001
< 5,000 RMB	54 (30.2%)	93 (57.4%)		
≥5,000 RMB	125 (69.8%)	69 (42.6%)		
**Sexual orientation**			< 0.001	1.000
Heterosexual/bisexual	18 (10.1%)	12 (7.4%)		
Homosexual	161 (89.9%)	150 (92.6%)		

### Change in Rush Popper usage

Before PEP usage, the proportion of individuals using Rush Popper at a frequency of ≥1 time/week was 17.6%. During PEP usage, this proportion increased to 23.8% (RR = 1.350, 95% CI: 1.002–1.819). After PEP usage, this proportion further rose to 29.6% (RR = 1.297, 95% CI: 1.104–1.524). Individuals with homosexual orientation experienced significant changes in Rush Popper usage during PEP usage (RR = 1.377, 95% CI: 1.011–1.877) and after PEP usage (RR = 1.323, 95% CI: 1.121–1.562) compared to before PEP usage. Among individuals aged 19–29 years (RR = 1.324, 95% CI: 1.068–1.642), unmarried (RR = 1.356, 95% CI: 1.130–1.628), registered residence in Qingdao (RR = 1.328, 95% CI: 1.094–1.612), with a college or higher degree (RR = 1.320, 95% CI: 1.107–1.570), and with an average monthly income < 5,000 RMB (RR = 1.528, 95% CI: 1.173–1.990), the proportion increased after PEP usage compared to before PEP usage. Additionally, changes in other subgroups were not significant (Table 2).

**Table 2:** Changes in Rush Popper usage.

**Group**	**Prevalence (%)**			**RR (95%CI)**	
	**Before PEP**	**During PEP**	**After PEP**	**During PEP vs. before PEP**	**After PEP vs. before PEP**
Total	17.6%	23.8%	29.6%	1.350 (1.002–1.819)^*^	1.297 (1.104–1.524)^**^
**Age group**					
19–29	18.3%	24.4%	32.1%	1.334 (0.903–1.971)	1.324 (1.068–1.642)^*^
30–39	17.6%	24.6%	27.6%	1.393 (0.824–2.350)	1.251 (0.948–1.652)
≥40	13.5%	19.9%	27.3%	1.417 (0.519–3.870)	1.421 (0.835–2.417)
**Marital status**					
Unmarried	17.2%	23.7%	31.6%	1.381 (0.976–1.954)	1.356 (1.130–1.628)^**^
Married/divorced/widowed	18.8%	23.8%	23.3%	1.265 (0.705–2.270)	1.112 (0.783–1.577)
**Registered residence**					
Qingdao	17.8%	24.3%	31.4%	1.370 (0.950–1.970)	1.328 (1.094–1.612)^**^
Others	17.2%	22.7%	26.2%	1.316 (0.784–2.210)	1.230 (0.923–1.648)
**Education**					
High school or lower	15.0%	24.7%	20.0%	1.645 (0.858–3.156)	1.150 (0.757–1.760)
College or higher	18.4%	23.5%	31.9%	1.277 (0.913–1.786)	1.320 (1.107–1.570)^**^
**Whether having a full-time job**					
Yes	18.4%	25.1%	31.4%	1.363 (0.996–1.866)	1.306 (1.103–1.546)^**^
No	13.2%	16.0%	19.2%	1.211 (0.474–3.095)	1.207 (0.715–2.037)
**Average monthly income**					
< 5,000 RMB	14.3%	22.9%	33.3%	1.600 (0.991–2.584)	1.528 (1.173–1.990)^**^
≥5,000 RMB	20.8%	24.4%	28.0%	1.172 (0.802–1.712)	1.160 (0.948–1.420)
**Sexual orientation**					
Heterosexual/bisexual	16.1%	16.7%	16.7%	1.033 (0.333–3.210)	1.017 (0.529–1.955)
Homosexual	17.7%	24.4%	31.1%	1.377 (1.011–1.877)^*^	1.323 (1.121–1.562)^***^

Risk ratio calculated using generalized estimating model fitting Poisson regression.

PEP, post-exposure prophylaxis; RR, risk ratio; CI, confidential interval.

^*^P < 0.05; ^**^P < 0.01; ^***^P < 0.001.

### Change in temporary sexual partners

Before PEP usage, 88.7% had ≥3 temporary sexual partners in the last 6 months. During PEP usage, this proportion was 91.8%, showing no significant change compared to before PEP usage (*P* > 0.05). However, subgroup analysis indicated a significant increase in this proportion among individuals with a college or higher degree during PEP usage (RR = 1.063, 95% CI: 1.002–1.128), rising from 86.6% to 99.3%. After PEP usage, the proportion of individuals with ≥3 temporary sexual partners in the last 6 months increased to 97.8% (RR = 1.056, 95% CI: 1.032–1.080). Notably, individuals aged 19–29 years (RR = 1.067, 95% CI: 1.029–1.106), unmarried (RR = 1.060, 95% CI: 1.031–1.091), registered in Qingdao (RR = 1.067, 95% CI: 1.039–1.096), with a college or higher degree (RR = 1.071, 95% CI: 1.045–1.098), with an average monthly income < 5,000 RMB (RR = 1.074, 95% CI: 1.040–1.114), and those with homosexual orientation (RR = 1.060, 95% CI: 1.034–1.087) experienced a proportion increase exceeding 10% (Table 3).

**Table 3:** Changes in temporary sexual partners.

**Group**	**Prevalence (%)**			**RR (95%CI)**	
	**Before PEP**	**During PEP**	**After PEP**	**During PEP vs. before PEP**	**After PEP vs. before PEP**
Total	87.7%	91.8%	97.8%	1.047 (0.995–1.102)	1.056 (1.032–1.080)^***^
**Age group**					
19–29	84.7%	88.1%	96.3%	1.040 (0.960–1.127)	1.067 (1.029–1.106)^***^
30–39	91.2%	97.5%	98.7%	1.069 (1.000–1.143)	1.040 (1.010–1.080)^*^
≥40	94.6%	91.5%	100.0%	0.967 (0.861–1.090)	1.030 (0.989–1.070)
**Marital status**					
Unmarried	86.3%	89.9%	97.1%	1.041 (0.977–1.110)	1.060 (1.031–1.091)^***^
Married/divorced/widowed	91.8%	97.6%	100.0%	1.064 (0.990–1.143)	1.044 (1.011–1.078)^**^
**Registered residence**					
Qingdao	87.2%	91.4%	99.2%	1.050 (0.984–1.120)	1.067 (1.039–1.096)^***^
Others	88.8%	92.4%	95.1%	1.041 (0.959–1.130)	1.035 (0.991–1.080)
**Education**					
High school or lower	91.2%	90.9%	91.4%	0.996 (0.903–1.100)	1.001 (0.942–1.060)
College or higher	86.6%	92.0%	99.3%	1.063 (1.002–1.128)^*^	1.071 (1.045–1.098)^***^
**Whether having a full-time job**					
Yes	88.2%	92.1%	98.0%	1.044 (0.989–1.102)	1.054 (1.029–1.080)^***^
No	84.9%	90.0%	96.2%	1.060 (0.916–1.230)	1.064 (0.994–1.140)
**Average monthly income**					
< 5,000 RMB	85.1%	91.4%	98.1%	1.074 (0.990–1.165)	1.074 (1.040–1.114)^***^
≥5,000 RMB	90.2%	92.0%	97.6%	1.021 (0.958–1.088)	1.040 (1.011–1.070)^**^
**Sexual orientation**					
Heterosexual/bisexual	96.8%	96.7%	100.0%	0.999 (0.911–1.100)	1.017 (0.984–1.050)
Homosexual	86.8%	91.3%	97.5%	1.052 (0.996–1.112)	1.060 (1.034–1.087)^***^

Risk ratio calculated using generalized estimating model fitting Poisson regression.

PEP, post-exposure prophylaxis; RR, risk ratio; CI, confidential interval.

^*^P < 0.05; ^**^P < 0.01; ^***^P < 0.001.

### Change in commercial sexual partners

Before PEP usage, 10.9% of participants had ≥3 commercial sexual partners in the last 6 months. During PEP usage, this proportion increased to 17.6% (RR = 1.622, 95% CI: 1.108–2.374). Significant changes were observed in subgroups such as unmarried (RR = 1.525, 95% CI: 1.012–2.299), non-registered in Qingdao (RR = 2.145, 95% CI: 1.062–4.329), with a college or higher degree (RR = 1.589, 95% CI: 1.024–2.466), without a full-time occupation (RR = 2.968, 95% CI: 1.153–7.638), an average monthly income ≥5,000 RMB (RR = 1.990, 95% CI: 1.148–3.450), and individuals with homosexual orientation (RR = 1.623, 95% CI: 1.099–2.399), while changes in other subgroups were not statistically significant during PEP usage. After PEP usage, this proportion further increased to 21.8% (RR = 1.417, 95% CI: 1.153–1.741). Except for those aged ≥40 years, with high school education or lower, with an average monthly income < 5,000 RMB, and reporting heterosexual/bisexual orientation, proportions in all other subgroups significantly increased compared to before PEP usage. Among them, individuals aged 30–39 years (RR = 1.939, 95% CI: 1.189–3.160), married/divorced/widowed (RR = 1.886, 95% CI: 1.127–3.157), and without a full-time occupation (RR = 1.806, 95% CI: 1.088–2.999) experienced a larger increase in proportions (Table 4).

**Table 4:** Changes in commercial sexual partners.

**Group**	**Prevalence (%)**			**RR (95%CI)**	
	**Before PEP**	**During PEP**	**After PEP**	**During PEP vs. before PEP**	**After PEP vs. before PEP**
Total	10.9%	17.6%	21.8%	1.622 (1.108–2.374)^*^	1.417 (1.153–1.741)^***^
**Age group**					
19–29	13.9%	21.0%	23.5%	1.517 (0.970–2.372)	1.301 (1.002–1.689)^*^
30–39	4.9%	11.9%	18.4%	2.420 (0.903–6.490)	1.939 (1.189–3.160)^**^
≥40	10.8%	19.1%	27.3%	1.771 (0.592–5.301)	1.588 (0.894–2.822)
**Marital status**					
Unmarried	12.5%	19.1%	22.1%	1.525 (1.012–2.299)^*^	1.328 (1.059–1.667)^*^
Married/divorced/widowed	5.9%	13.1%	20.9%	2.226 (0.808–6.132)	1.886 (1.127–3.157)^*^
**Registered residence**					
Qingdao	12.0%	17.1%	22.0%	1.426 (0.903–2.253)	1.355 (1.060–1.732)^*^
Others	8.6%	18.5%	21.3%	2.145 (1.062–4.329)^*^	1.572 (1.073–2.304)^*^
**Education**					
High school or lower	11.2%	19.5%	20.0%	1.732 (0.806–3.721)	1.333 (0.848–2.096)
College or higher	10.7%	17.0%	22.2%	1.589 (1.024–2.466)^*^	1.439 (1.141–1.816)^**^
**Whether having a full-time job**					
Yes	11.1%	15.8%	20.3%	1.423 (0.934–2.167)	1.350 (1.077–1.694)^**^
No	9.4%	28.0%	30.8%	2.968 (1.153–7.638)^*^	1.806 (1.088–2.999)^*^
**Average monthly income**					
< 5,000 RMB	12.5%	16.4%	22.2%	1.314 (0.760–2.270)	1.333 (0.968–1.840)
≥5,000 RMB	9.5%	18.4%	21.6%	1.990 (1.148–3.450)^*^	1.528 (1.147–2.036)^**^
**Sexual orientation**					
Heterosexual/bisexual	6.5%	10.0%	16.7%	1.550 (0.278–8.633)	1.607 (0.690–3.750)
Homosexual	11.3%	18.3%	22.4%	1.623 (1.099–2.399)^*^	1.407 (1.138–1.740)^**^

Risk ratio calculated using generalized estimating model fitting Poisson regression.

PEP, post-exposure prophylaxis; RR, risk ratio; CI, confidential interval.

^*^P < 0.05; ^**^P < 0.01; ^***^P < 0.001.

### Change in group sex

Before PEP usage, the proportion of individuals engaging in group sex more than five times in the last 6 months was 30.8%. During PEP usage, this proportion decreased to 21.4% (RR = 0.695, 95% CI: 0.537–0.900). Notably, individuals aged ≥40 years (RR = 0.437, 95% CI: 0.230–0.831), married/divorced/widowed (RR = 0.538, 95% CI: 0.324–0.891), with a college or higher degree (RR = 0.701, 95% CI: 0.521–0.943), with a full–time job (RR = 0.645, 95% CI: 0.487–0.855), and with an average monthly income ≥5,000 RMB (RR = 0.703, 95% CI: 0.511–0.966) experienced a larger decrease in proportions during PEP usage. After PEP usage, the proportion decreased to 21.2% (RR = 0.830, 95% CI: 0.706–0.976) compared to before PEP usage. Individuals aged ≥40 years (RR = 0.529, 95% CI: 0.305–0.919), with a college or higher degree (RR = 0.816, 95% CI: 0.677–0.983), with a full–time job (RR = 0.796, 95% CI: 0.666–0.952), with an average monthly income ≥5000 RMB (RR = 0.789, 95% CI: 0.649–0.960), and reporting a homosexual orientation (RR = 0.831, 95% CI: 0.698–0.990) experienced a significant decrease in proportions after PEP usage, while other subgroups showed no significant changes (Table 5).

**Table 5:** Changes in group sex.

**Group**	**Prevalence (%)**			**RR (95%CI)**	
	**Before PEP**	**During PEP**	**After PEP**	**During PEP vs. before PEP**	**After PEP vs. before PEP**
Total	30.8%	21.4%	21.2%	0.695 (0.537–0.900)^**^	0.830 (0.706–0.976)^*^
**Age group**					
19–29	26.2%	19.9%	21.0%	0.758 (0.520–1.100)	0.894 (0.703–1.140)
30–39	33.3%	23.7%	23.7%	0.712 (0.466–1.088)	0.843 (0.660–1.076)
≥40	48.6%	21.3%	13.6%	0.437 (0.230–0.831)^*^	0.529 (0.305–0.919)^*^
**Marital status**					
Unmarried	28.5%	21.8%	20.6%	0.764 (0.565–1.034)	0.850 (0.702–1.029)
Married/divorced/widowed	37.6%	20.2%	23.3%	0.538 (0.324–0.891)^*^	0.786 (0.580–1.065)
**Registered residence**					
Qingdao	31.1%	22.5%	22.9%	0.724 (0.530–0.989)^*^	0.858 (0.708–1.039)
Others	30.2%	19.3%	18.0%	0.641 (0.404–1.014)	0.773 (0.572–1.040)
**Education**					
High school or less	32.5%	22.1%	25.7%	0.679 (0.402–1.150)	0.889 (0.644–1.228)
College or higher	30.3%	21.2%	20.1%	0.701 (0.521–0.943)^*^	0.816 (0.677–0.983)^*^
**Whether having a full-time job**					
Yes	31.9%	20.6%	20.3%	0.645 (0.487–0.855)^**^	0.796 (0.666–0.952)^*^
No	24.5%	26.0%	26.9%	1.060 (0.545–2.060)	1.048 (0.706–1.555)
**Average monthly income**					
< 5,000 RMB	26.8%	17.1%	20.4%	0.640 (0.411–0.995)^*^	0.872 (0.651–1.168)
≥5,000 RMB	34.7%	24.4%	21.6%	0.703 (0.511–0.966)^*^	0.789 (0.649–0.960)^*^
**Sexual orientation**					
Heterosexual/bisexual	41.9%	23.3%	27.8%	0.556 (0.258–1.200)	0.814 (0.531–1.250)
Homosexual	29.7%	21.2%	20.5%	0.715 (0.544–0.941)^*^	0.831 (0.698–0.990)^*^

Risk ratio calculated using generalized estimating model fitting Poisson regression.

PEP, post-exposure prophylaxis; RR, risk ratio; CI, confidential interval.

^*^P < 0.05; ^**^P < 0.01.

## Discussion

This study investigated changes in risk behaviors among MSM across three stages: before, during, and after PEP usage. The findings revealed significant changes in risk behaviors among MSM after PEP usage. Notable increases were observed in Rush Popper usage, temporary sexual partners, and commercial sexual partners. These changes could be attributed to either the utilization of PEP or broader behavioral shifts within the MSM community. Regardless of the underlying cause, this phenomenon raises concerns for HIV prevention and control among MSM. If PEP usage contributes to increased risk behaviors among MSM, it implies a potential risk compensation linked to PEP usage. Further research is essential to elucidate whether promoting PEP is genuinely beneficial for HIV prevention in MSM. However, given the absence of a parallel control group for those not using PEP in this study, the observed changes may also reflect general behavioral shifts within the entire population during the study period, potentially unrelated to PEP utilization. If this is the case, it underscores the escalating risk behaviors among MSM in Qingdao.

The World Drug Report 2022 indicated a global rise in the use of recreational drugs and associated adverse consequences (19). Due to their stimulant and hallucinogenic effects, the use of recreational drugs could potentially facilitate high-risk sexual behaviors among MSM, elevating the risk of HIV transmission in this population (20–22). Recreational drug use exhibited regional variations influenced by legal systems, economic status, and drug cultures (23). In China, Rush Popper emerged as the primary recreational drug among MSM, readily accessible and easily obtained through the Internet. In this study, the proportion of MSM using Rush Popper ≥1 time/week significantly increased both during and after PEP usage, suggesting that PEP may contribute to heightened Rush Popper use in this population. This finding aligns with the observed increase in Rush Popper usage among MSM in China (24, 25), implying a broader shift within the MSM community. The HIV interventions in China primarily targeted individuals injecting traditional drugs, with limited emphasis on recreational drug users (26). These results suggested an urgent need to strengthen the intervention of recreational drug use such as Rush Popper.

As a sexual minority, some MSM were influenced by the traditional Chinese marriage culture, leading them to enter into marriages (27). This subgroup acts as a bridge across from homosexual to heterosexual transmission of HIV, thereby contributing to the expansion of HIV transmission. Shandong Province, the birthplace of Confucianism, is deeply influenced by traditional values and exhibits a pronounced stigmatization of homosexual orientation (28). In this study, individuals who were married/divorced/widowed constituted approximately one-fourth of the population. The proportion of temporary and commercial sexual partners in this group increased significantly after PEP usage, potentially escalating the risk of HIV transmission to females. Due to social discrimination and privacy concerns, some MSM reported a heterosexual/bisexual orientation. In contrast, those who reported homosexual orientation had a higher self-identification. The Rush Popper usage, temporary sexual partners, and commercial sexual partners in this group increased significantly after PEP usage, indicating an increasing risk of HIV infection.

Compared to traditional HIV prevention measures like condom use, PEP represented a relatively recent and underutilized biomedical HIV preventive measure (29, 30). It was not until 2020 that the Chinese CDC issued guidelines on PEP (31). The high cost of PEP, without compensatory measures, increased the likelihood that MSM of higher socioeconomic status would be aware of and utilize PEP (32). In this study, a notable increase in the proportion of temporary and commercial sexual partners was observed after PEP usage among individuals with higher education levels, full-time professionals, and those with higher monthly incomes. Furthermore, younger MSM, known for being more sexually active and less risk-averse, exhibited significant increases in Rush Popper usage, temporary sexual partners, and commercial sexual partners after PEP usage. These findings implied the importance of accompanying PEP promotion with intensified HIV prevention education for MSM, with a specific focus on the above vulnerable groups.

The present study demonstrated a significant decrease in the proportion of individuals engaging in group sex >5 times in the last 6 months both during and after PEP usage, compared with before PEP usage. In part, it might be explained by the fact that PEP users realized the risks of group sex and consequently curtailed such activities. But since no further investigations were conducted in this study, there was no definite evidence to support this speculation. Alternatively, this decline could be linked to various restrictions imposed during the COVID-19 epidemic, such as quarantine and traffic restrictions, which hindered MSM from gathering and consequently led to a reduction in group sex within this population. In particular, older individuals were more significantly impacted by the lockdown measures during the COVID-19 epidemic, due to their preference for offline searching for sexual partners, which was similar to the findings of Wang et al. (33). In addition, the proportion decreased among MSM of different economic status, with a more substantial percentage decrease among those with higher monthly incomes. After PEP usage, the proportion of those with higher monthly incomes remained decreased, while no significant changes were observed among those with lower monthly incomes. This discrepancy could be related to a higher proportion of loss to follow-up among individuals with lower monthly incomes, potentially influencing the results.

There are several limitations in this study. Firstly, the absence of a control group comprising MSM not using PEP is a limitation. Instead, the study compares the behaviors of MSM before, during, and after PEP usage. It remains uncertain whether individuals not using PEP experienced any changes during this period. Secondly, this study was conducted during the COVID-19 pandemic, many participants were lost to follow-up due to the lockdown measures, potentially impacting the results. Moreover, given the small sample size and limited representativeness, the results need to be generalized with caution. Despite the limitations of this study, it provides implications for future research. We found that PEP utilization had the potential to impact risk behaviors of MSM, but this possibility could not yet be clarified due to limitations of the study. It is suggested that there is a need for additional research in this area. Moreover, this study found that risk behaviors of MSM in Qingdao were severe, and exploring how to reduce risk behaviors of MSM is also important in this area.

## Conclusion

The utilization of PEP as a novel biomedical preventive measure could potentially contribute to risk compensation among MSM. After PEP usage, MSM might experience an increase in Rush Popper usage, temporary sexual partners, and commercial sexual partners, accompanied by a potential decrease in the frequency of group sex. These observed changes varied by sociodemographic characteristics. Subsequent research endeavors are imperative to elucidate the nuanced impact of PEP usage on MSM and to formulate targeted, effective HIV prevention programs.

## Data availability statement

The data analyzed in this study is subject to the following licenses/restrictions: The datasets presented in this article are not readily available because of privacy and ethical concerns. Requests to access the datasets should be directed to the corresponding author. Requests to access these datasets should be directed to DL, lidongmin@chinaaids.cn.

## Ethics statement

The studies involving humans were approved by the Ethics Committee of National Center for AIDS/STD Control and Prevention, Chinese Center for Disease Control and Prevention. The studies were conducted in accordance with the local legislation and institutional requirements. The participants provided their written informed consent to participate in this study.

## Author contributions

RS: Conceptualization, Data curation, Formal analysis, Investigation, Methodology, Writing – original draft, Project administration, Writing – review & editing. YL: Data curation, Investigation, Supervision, Writing – review & editing, Software. PL: Data curation, Project administration, Resources, Supervision, Writing – review & editing. LG: Methodology, Project administration, Supervision, Validation, Writing – review & editing. ML: Investigation, Project administration, Supervision, Writing – review & editing. YF: Investigation, Project administration, Supervision, Validation, Writing – review & editing. XS: Investigation, Supervision, Validation, Writing – review & editing, Resources. HT: Conceptualization, Methodology, Project administration, Resources, Writing – review & editing, Software. DL: Conceptualization, Funding acquisition, Investigation, Methodology, Project administration, Resources, Supervision, Writing – review & editing.
